# *In silico* drug screening and potential target identification for hepatocellular carcinoma using support vector machine

**DOI:** 10.1186/1753-6561-6-S6-P16

**Published:** 2012-10-01

**Authors:** Wu-Lung R Yang, Yu-En Lee, Ming-Huang Chen, Yu-Wen Liu, Pei-Ying Lee, Kun-Mao Chao, Chi-Ying F Huang

**Affiliations:** 1 Department of Computer Science and Information Engineering, National Taiwan University, Taipei, Taiwan; 2 lnstitute of Biotechnology in Medicine, National Yang-Ming University, Taipei, Taiwan; 3 lnstitute of Clinical Medicine, National Yang-Ming University, Taipei, Taiwan; 4 Division of Hematology and Oncology, Department of Medicine, Taipei Veterans General Hospital, Taipei, Taiwan; 5 Graduate Institute of Biomedical Electronic and Bioinformatics, National Taiwan University, Taipei, Taiwan; 6 Institute of Biopharmaceutical Sciences, National Yang-Ming University, Taipei, Taiwan

## 

Hepatocellular carcinoma (HCC) is a severe liver malignancy with few drug treatment options. Drug screening using FDA-approved drugs will provide a fast track in clinical trials if drugs are found effective against HCC. The Connectivity Map (cmap), a large repository of chemical-induced gene expression profiles, provides the opportunity of analyzing drug property with the expression. A support vector machine (SVM) was utilized to classify the effectiveness of drugs against HCC using gene expression profiles in cmap. The classification results will help us to identify significant chemical-sensitivity genes, and to predict the effectiveness of remaining chemicals in cmap, with a prioritized listing for biological verification. The cell viability of four HCC cell lines treated with 146 chemicals was conducted. The SVM successfully classified the effectiveness of chemicals with an average area under the receiver operating curve of 0.9. Chemical sensitivity genes which are possible HCC therapeutic targets, such as *MT1E*, *MYC* and *GADD45B*, were identified with opposite signs of gene differential changes compared with reported HCC patient samples. Several known HCC inhibitors, such as geldanamycin, alvespimycin (histone deacetylase inhibitors) and doxorubicin (chemotherapy drug), were predicted to be effective. Seven out of 23 predicted drugs were cardiac glycosides, suggesting a close link of these drugs to the inhibition of HCC. The study demonstrates a strategy of *in silico* drug screening using a large repository of microarrays based on initial in vitro drug screening results. The biological verification result can serve as a feedback into the process for the development of a more accurate chemical sensitivity model.

